# Retrospective Study of the Reasons and Time Involved for Dental Providers' Medical Consults

**DOI:** 10.3389/fdgth.2022.838538

**Published:** 2022-05-12

**Authors:** Shuning Li, Karmen S. Williams, Jayanth Kumar Medam, Jay S. Patel, Theresa Gonzalez, Thankam P. Thyvalikakath

**Affiliations:** ^1^Dental Informatics, Indiana University School of Dentistry, Indianapolis, IN, United States; ^2^Fairbanks School of Public Health, Indiana University-Purdue University Indianapolis (IUPUI), Indianapolis, IN, United States; ^3^Population Health Informatics, Graduate School of Public Health and Health Policy, City University of New York, New York, NY, United States; ^4^ELLKAY LLC, Elmwood Park, NJ, United States; ^5^Temple University, Philadelphia, PA, United States; ^6^Center for Biomedical Informatics, Regenstrief Institute, Inc., Indianapolis, IN, United States

**Keywords:** medical consult, axiUm, regional health information exchange, electronic health record, electronic dental record, dental informatics, health informatics, patient safety

## Abstract

**Background:**

Patient-reported medical histories and medical consults are primary approaches to obtaining patients' medical histories in dental settings. While patient-reported medical histories are reported to have inconsistencies, sparse information exists regarding the completeness of medical providers' responses to dental providers' medical consults. This study examined records from a predoctoral dental student clinic to determine the reasons for medical consults; the medical information requested, the completeness of returned responses, and the time taken to receive answers for medical consult requests.

**Methods:**

A random sample of 240 medical consult requests for 179 distinct patients were selected from patient encounters between 1 January 2015 and 31 December 2017. Descriptive statistics and summaries were calculated to determine the reasons for the consult, the type of information requested and returned, and the time interval for each consult.

**Results:**

The top two reasons for medical consults were to obtain more information (46.1%) and seek medical approval to proceed with treatment (30.3%). Laboratory and diagnostic reports (56.3%), recommendations/medical clearances (39.6%), medication information (38.3%), and current medical conditions (19.2%) were the frequent requests. However, medical providers responded fewer times to dental providers' laboratory and diagnostic report requests (41.3%), recommendations/medical clearances (19.2%), and current medical conditions (13.3%). While 86% of consults were returned in 30 days and 14% were completed after 30 days.

**Conclusions:**

The primary reasons for dental providers' medical consults are to obtain patient information and seek recommendations for dental care. Laboratory/diagnostic reports, current medical conditions, medication history, or modifications constituted the frequently requested information. Precautions for dental procedures, antibiotic prophylaxis, and contraindications included reasons to seek medical providers' recommendations. The results also highlight the challenges they experience, such as requiring multiple attempts to contact medical providers, the incompleteness of information shared, and the delays experienced in completing at least 25% of the consults.

**Practical Implications:**

The study results call attention to the importance of interdisciplinary care to provide optimum dental care and the necessity to establish systems such as integrated electronic dental record-electronic health record systems and health information exchanges to improve information sharing and communication between dental and medical providers.

## Introduction

Patient safety is of utmost importance in all health care settings, including dental care. To avoid potential harm (1, 2) and improve success during dental treatment (3, 4), dental providers must obtain up-to-date medical and medication history from each patient. In the US, approximately 45% of the adult population has at least one chronic disease (5), and 26–33% of the adult population and 45% of adults over 65 years of age have multiple chronic conditions (MCC) (6, 7). Globally, at least one in three adults has MCC. Furthermore, the frequency of MCC is estimated to rise exponentially during the next two decades (7). Therefore, providing safe and effective dental treatment for all patients, especially those with MCC, requires access to current and accurate medical records for dental providers to avoid adverse patient outcomes. Dental providers often seek medical consults to fill gaps in patient-reported medical history. However, little has been published regarding the time from request to response and the totality of the medical information returned.

In dental practices, medical information is gathered in two ways—through patient-reported medical history and consults with the patients' medical providers. Patient-reported medical histories are collected through health questionnaires administered before a dental examination. Studies that evaluated patient-reported medical conditions, medication history, and other health-related histories reported varied concordance and reliability compared to medical records (8–11). For instance, dental patients reported certain medical conditions, such as myocardial infarction, stroke, and coronary artery disease, but omit other conditions, such as cardiomyopathy, atrial fibrillation, and carotid artery syndrome (8, 11). Studies have also reported inconsistencies in patients reporting common medical conditions such as diabetes, hypertension, and medication histories (12, 13). Therefore, patient-reported medical histories, especially for dental patients with MCC, are confirmed with their medical providers before dental treatment (14).

Medical consult is “the procedure of seeking the opinion of another health care provider in the development of management schemes” (15). Experts advise dental providers to consult the patients' physicians to obtain critical medical information such as patient medications, laboratory reports, and current medical condition statuses to adjust the medication regimen, treatment recommendations, and medical clearance before dental treatment (15–17). However, reports suggest continued challenges with contacting physician offices for medical consults, such as not obtaining information on time (18). In addition, except for expert opinions on the continuity of care, there is sparse information regarding the information dental clinicians seek during medical consults and to what extent medical providers respond to dental clinicians' requests.

The objectives of our retrospective study of medical consults at the Indiana University School of Dentistry (IUSD) were to determine: (1) the reasons for initiating medical consults, (2) the information dental providers requested, (3) the information the consulting physician shared in the returned medical consults, and (4) the time taken to complete the medical consults. The results of this study will determine the information dental providers require through medical consults and the extent to which medical providers furnish the dental providers' information requests.

## Materials and Methods

IUSD utilizes the electronic dental record system axiUm (Exan software, Las Vegas, Nevada, USA) to record patient care activities. When indicated for patient care, under the supervision of the clinic faculty, the pre-doctoral student dental providers (hereby referred to as dental providers) complete a medical consult form within axiUm under the clinic faculty's supervision. Following the clinic faculty's approval, the dental provider prints and faxes the medical consult form to the medical provider. The medical provider responds via return fax to the IUSD Office of Clinical Affairs, where the returned document is scanned into axiUm as pdf files, and the dental provider is notified of the returned medical consults. Based on the received information, dental providers will modify and/or proceed with the definitive treatment. If dental providers do not receive the requested information, they will continue contacting the medical providers' offices by phone and fax until they receive the information. In the meantime, the patients are on hold or receive symptomatic treatments, such as antibiotics and pain medication prescriptions, and not definitive treatments, such as tooth extractions and other dental procedures.

We performed a retrospective, cross-sectional study of medical consult requests initiated between 1 January 2015 and 31 December 2017, for patients 18 years and older. The clinical systems manager at IUSD selected the medical consults using the random generator function in MS Excel from the 4,586 medical consultations initiated during this three-year time. The scanned medical consults returned from physician offices were reviewed manually for data abstraction because the scanned files have low image quality and most of them also have handwriting responses. These situations made it difficult to automate the retrieval of pertinent information. Therefore, we must manually review all the files and abstract information. In this study, we reviewed medical consults until achieving data saturation with no new information being abstracted, a process followed in qualitative research (19). This study received exempt IRB approval from the Indiana University IRB (Protocol #1801003555).

### Development of Data Abstraction Guideline and Form

**Figure 1:**
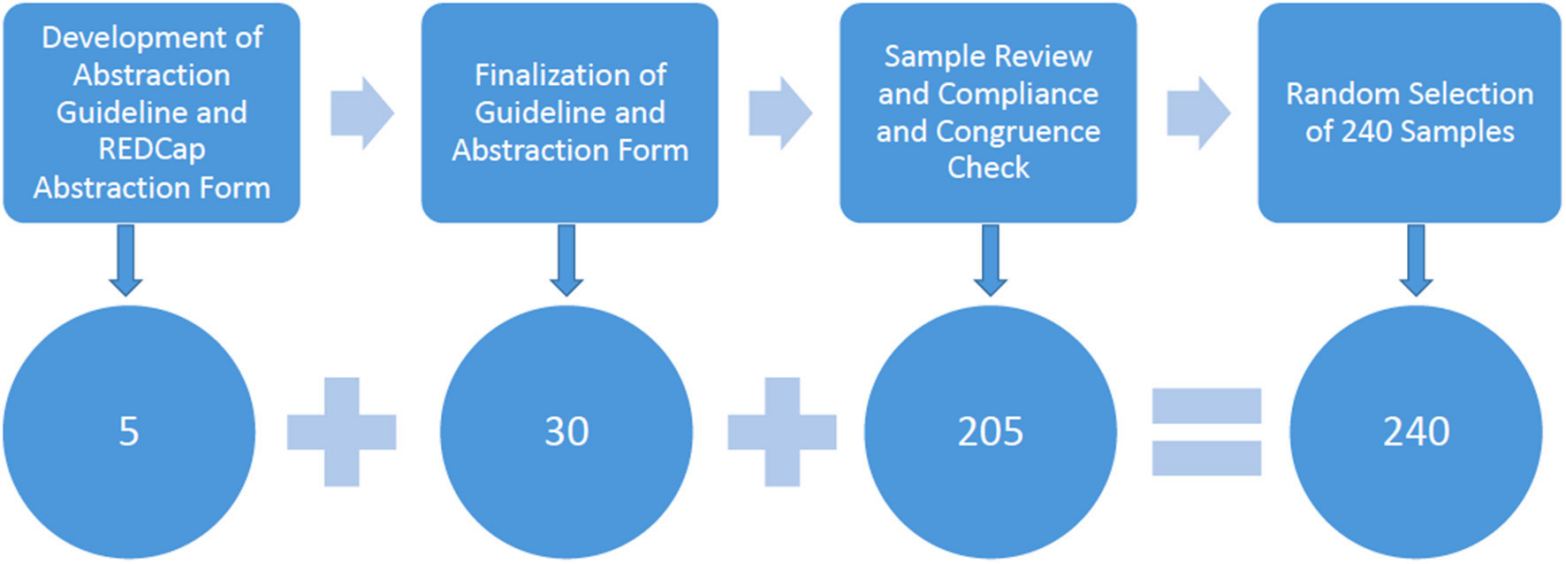
Flow diagram demonstrating the different steps during the medical consult review process (single column fitting image).

Figure 1 illustrates the steps in developing the data abstraction guideline and the subsequent review process. Two researchers developed an initial approach to abstract the data from axiUm based on prior literature (16, 17). The reviewers tested this initial guideline by abstracting relevant information from five medical consults belonging to five patients and entering them into a data entry form created in REDCap (Research electronic data capture) (Figure 1). REDCap is a workflow methodology and software solution designed for electronic data capture to support clinical and translational research (20).

Four team members independently reviewed another 30 consults to finalize the guideline and the data entry form in REDCap (Figure 1). Any inconsistencies in the review were discussed among the researchers and resolved. The researchers also discussed and verified the definitions of information categories, calculating age, and defined terms in the medical consult form. They defined 17 information items as described in Table 1, which included the following categories: (1) patient demographics, (2) reason(s) for the medical consults, (3) information dental providers requested, (4) information dental providers reported in the medical consults, (5) information medical providers reported in the returned medical consult form, (6) medical consult response time, and (7) miscellaneous information which cannot be assigned to the categories above.

**Table 1:** Data abstraction guidelines were developed and used to abstract information from the medical consult forms.

	**Information item**	**Description and example**
**Patient information**		
1	Record ID	
2	Age	*Patient age at time of encounter; difference between the date of birth and the date medical consult was sent to medical provider*
3	Gender	*Listed in the electronic dental record (i.e., Male, Female, Unknown)*
**Reason for medical consult**		
4	Reason for the medical consult	*Specific or implied reason written on the medical consult form*
5	Medical provider specialty	*i.e., Family Practice, Internal Medicine, Cardiology*
6	Medical provider address	*Private practice or facility address*
**Information reported by dental providers in the medical consult form**		
5	Dental conditions/diagnoses	*i.e., abscess, tooth pain*
6	Dental treatments	*i.e., course of treatment, type of treatment, etc*.
7	Medication to be used during dental treatment	*i.e., local anesthetic*
8	Medication to be used after dental treatment	*i.e., pain medication*
9	Medical conditions and medications as reported by the patient	*Any medical and medication information provided by the patient*
**Information requested by dental providers**		
10	Medication history of the patient	*i.e., pain medication, list of medication/s antihypertensive medication, etc*.
11	Medical condition(s) of the patient	*Current medical status, condition(s) status and laboratory and/or diagnostic reports (i.e., medical history, cardiac status, HbA1c, viral load, CD4 count, echocardiogram report, recommendations etc.)*
**Information reported by physicians in the returned medical consult form**		
12	Medical condition(s) of the patient	*Any medical information regarding a patient*'*s condition(s)*
13	Medication history	*Any medication(s) the patient is currently prescribed, for example, anticoagulant medication*
14	Contradictions for dental treatment	*recommendations and medical clearance (i.e., antibiotic prophylaxis, contraindications, medical clearance etc.)*
**Medical consult response time**		
15	Number of days taken for the medical provider's response to medical consult	*difference between the date of sending medical consult and date when medical consult was scanned into patient record (when available for dental provider to view information)*
**Miscellaneous**		
16	Complete patient health record provided	*Medical provider sent complete patient record without responding to dental provider*'*s questions*

Subsequently, the REDCap (20) data entry form was finalized to abstract information from the remaining consults until data saturation was achieved (Figure 1). Two reviewers (KW, JKM) verified the form for compliance with the data abstraction guideline and checked for congruence by checking three medical consults completed by each reviewer. Any inconsistencies were discussed among the researchers and resolved.

### Data Analysis

**Figure 2:**
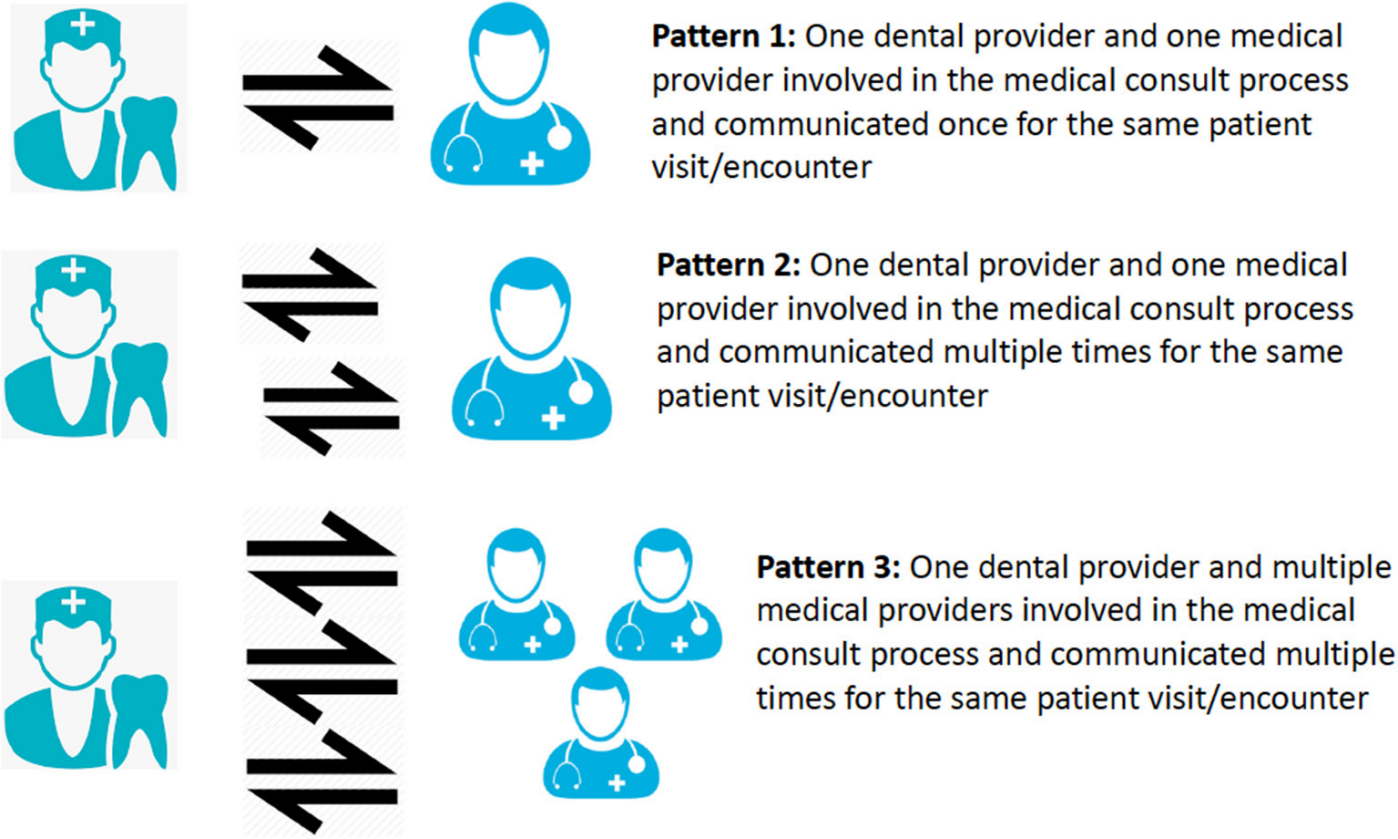
Three communication patterns between dental and medical providers.

Descriptive statistics were calculated for demographic information and medical consult response time (Table 1). For analysis purposes, a consult sent to one medical provider constituted one medical consult (Figure 2). A patient may have multiple medical consults if they see numerous medical providers or if one provider was contacted multiple times based on their underlying conditions. Therefore, medical consults were grouped based on the number of medical providers contacted and the number of times they were contacted. As for the collected information items, the reasons for medical consults were classified according to the reviewer guidelines (see Table 1); dental providers requests, and medical provider returned patient information was classified based on the information types defined in the guideline. Finally, we calculated the frequencies of different information types dental providers requested, the information reported in the medical consults; and the frequencies of different information types reported in the returned medical consult forms. Medical provider specialty and affiliation were also summarized to further describe the medical consults.

## Results

A total of 240 medical consults were reviewed to achieve saturation of information requested and shared between dental and medical providers. These consults were initiated for 179 patients aged 18 years or older by the IUSD dental providers in the pre-doctoral clinics. From the entire medical consults, 4,080 information items were abstracted.

Among the 240 medical consults, 131 consults (55%) followed Pattern 1, a single communication between one dental provider and one medical provider (Figure 2), and 61 (25%) consults involved multiple communications between one dental provider and one medical provider (Pattern 2) to complete the consults. Forty-eight consults (20%) involved one dental provider having multiple communications with more than one medical provider (Pattern 3).

Among the 179 patients, 131 patients (73%) had their medical consults completed with a single consult request per patient. Thirty-six patients (20%) had consults completed with two consult requests per patient. Eleven patients (6%) had three consult requests per patient, and one (1%) had more than three consults.

### Patient Characteristics

Among the 179 patients, 94 (52.5%) patients were males, 84 (46.9%) were females, and 1 (0.55%) patient was reported as other gender. The mean age was 61.3 years ± 15.25 (SD).

### Reasons for Medical Consults

Table 2 lists the four major reasons for dental providers to seek medical consults, in which requesting “*additional medical information that the patient could not provide”* ranked the highest (46.1% of medical consults). In total, 436 reasons were listed for the 240 medical consults because more than one reason was listed for 156 consults (116 had two reasons and 40 had three reasons).

**Table 2:** Reasons for dental providers to seek medical consults for 179 patients.

**Reason for medical consults**	Number (%)^*^
Patient provided some information leading to the Dentist's medical consult initiation for more information.	201 (46.1)
Dental provider wanted the physician's confirmation for proceeding with the planned dental treatment.	132 (30.3)
Dental provider wanted to confirm the patient provided medical and medication information.	101 (23.2)
Patient could not provide any information leading to the Dentist's medical consult initiation.	1 (0.2)
Dental provider initiated the medical consult with the suggestion from the previously contacted physician.	1 (0.2)
Total	436 (100)

* *Total number is more than 240 due to the presence of multiple reasons for one consult*.

### Information Dental Providers Requested in the Medical Consults

**Figure 3:**
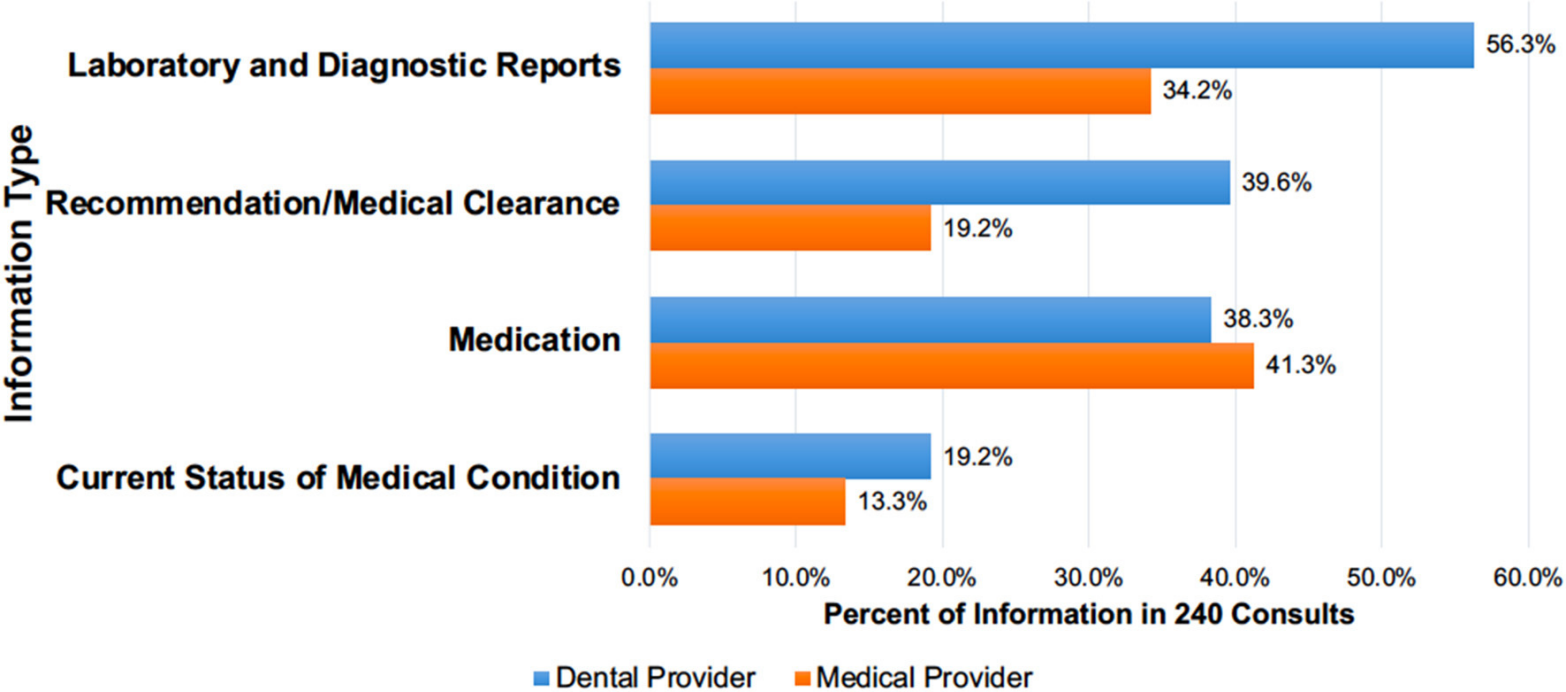
The percentage of different information types* dental providers requested in the 240 medical consults for 179 patients and returned by medical providers (2-column fitting image). *Values do not add up to 240 (or 100%) due to the presence of multiple information requests in one form.

The dental providers requested patients' laboratory values and diagnostic written reports (56.3%), recommendations and/or medical clearances (39.6%), medication information (38.3%), and current medical conditions and status (19.2%) (Figure 3).

Of the 135 consults (56.3%) that requested laboratory and diagnostic written reports, HbA1c values (46.8%) were requested followed by other lab values (37%), and INR (International normalized ratio)/prothrombin time (14.1%) (Table 3). Whereas, for the 95 consults (39.6%) that requested recommendations/medical clearance, 42.1% asked for precautions to dental procedures, 30.5% for contraindications, 20% for general recommendations regarding planned treatment, and 10.5% regarding the use of local anesthetic with vasoconstrictor. Of the 92 consults (38.3%) that requested medication information, 46.7% of the requests asked for recommendations regarding the need for antibiotic prophylaxis during dental care (Table 3). Internal medicine was the most frequently consulted medical specialty regarding antibiotic prophylaxis (34.9%, 15 out of 43 antibiotic prophylaxis requests), followed by orthopedics (27.9%, 12 out of 43) and family medicine (25.6%, 11 out of 43). Other medication-related requests were 27.2% for the medication lists, 14.1% for recommendations to suggest or modify medications, and 12% for the anticoagulant medication (Table 3).

**Table 3:** The specific types of information (aside from medical conditions), dental providers (DP) requested vs. medical providers (MP) returned within the 240 medical consults.

**Laboratory and diagnostic reports**	**DP requests**		**MP returns**		**Recommendations**	**DP requests**		**MP returns**		**Medication/s related**	**DP requests**		**MP returns**	
	***N =* 135 (56.3%)**	**^†^ (%)**	***N =* 82 (34.2%)**	**^†^ (%)**		***N =* 95 (39.6%)**	**^†^ (%)**	***N =* 46 (19.2%)**	**^†^ (%)**		***N =* 92 (38.3%)**	**^†^ (%)**	***N =* 99 (41.3%)**	**^†^ (%)**
HbA1c	63	(46.7)	34	(41.5)	Precautions to dental procedures	40	(42.1)	14	(30.4)	Antibiotic prophylaxis	43	(46.7)	46	(46.5)
Other lab values^*^	50	(37)	22	(26.8)	Contraindications	29	(30.5)	10	(21.7)	Medication list	25	(27.2)	34	(34.3)
INR/Prothrombin time	19	(14.1)	9	(11)	General recommendations for planned treatments	19	(20)	19	(41.3)	Suggest or modify medications	13	(14.1)	8	(8.1)
Blood pressure	16	(11.9)	17	(20.7)										
CD4 counts/Viral load	15	(11.1)	14	(17.1)										
Other diagnostic reports	9	(6.7)	6	(7.3)	Local anesthesia with vasoconstrictor	10	(10.5)	6	(13)	Anticoagulant medication	11	(12)	14	(14.1)
Echocardiogram	8	(5.9)	3	(3.7)										
Ejection fraction	3	(2.2)	0	(0.0)										

† *Does not add up to 100% because each medical consult can have multiple requests and returns*.

* *Complete blood count, liver function test, tuberculosis test, etc*.

### Information Dental Providers Reported in the Medical Consults

In 97% of the medical consults, the dental providers included the planned dental treatment such as administration of local anesthetic (67%), tooth extractions (60%), scaling and root planning (51.3%), restorations (44.6%), and post-operative pain medications (2.5%). In 8.3% of the consults, the dental providers also reported the patients' dental conditions.

### Information Medical Providers Returned in the Medical Consults

Of the 240 medical consults, 17.5% of the consults did not return requested information (shared blank pages) and 2.6% of the consults had only the medical provider's signature with no information. In 46% of the consults, the medical providers shared additional information to the requested information. The medical providers sent the complete medical record in 24% of the returned consults, however, 10% of these consults did not specifically address the dental provider's requests. Medical providers responded to requests for medication information (41.3%), laboratory and diagnostic reports (34.2%), requests for recommendations or medical clearance (19.2%), and current medical status (13.3%) (Figure 3).

Of the 82 consults (34.2%) that responded with laboratory and diagnostic written reports, 41.5% included responses for HbA1c values, 26.8% responded for other lab values, and 20.7% responded for blood pressure values (Table 3). Regarding the 95 consults (39.6%) for recommendations/medical clearance, 41.3% of the consults included recommendations for the planned treatment, 30.4% included precautions to dental procedures, 21.7% shared contraindications, and 13% advised regarding the use of local anesthetic with vasoconstrictor. Table 3 also lists the percentage of consults for which medical providers responded with recommendations regarding the need for antibiotic prophylaxis during dental care (46.5%), medication list (34.3%), suggestions or modifications for medications (8.1%), and anticoagulant medications (14.1%) (Table 3).

The most frequently consulted medical specialties were family medicine (33.8%), internal medicine (24.6%), cardiology (10.4%), orthopedics (6.3%), and infectious diseases (4.2%). The remaining 20.7% were sent to other 20 different types of medical specialties such as endocrinology, neurology, and sports medicine. These medical providers were associated with the Indiana University Healthcare system (23.8%) or one of two large community hospital systems (11.3 and 9.6%, respectively). The remaining 55.3% were associated with unique health facilities in Indiana and other states.

### Time Taken to Complete Medical Consults

The medical consult requests were returned on an average in 19.6 ± 36.6 days with 57% of requests returned within 10 days (Table 4). It took over 30 days for 13.8% of the medical consults to be returned to the dental provider and seven of the medical consults were returned more than 100 days after the request was made. Among the 179 patients, 16.8% of them (30 of 179 patients) had at least one consult returned more than 30 days after the request.

**Table 4:** Number of days taken to complete medical consult (*N* = 240).

**Days**	**Number of consults**	**Percent**
0–10	137	57.1%
11–20	45	18.8%
21–30	25	10.4%
31–40	4	1.7%
41–50	8	3.3%
51–60	8	3.3%
61–70	3	1.3%
71+	10	4.2%

## Discussion

Providing expert care for patients requires coordination, communication, and transparency among all care providers. In this study, from one institute that performed an in-depth analysis of dental providers' medical consults, the main reasons for the medical consults were to obtain additional patient-related information and to seek recommendations or medical clearance. Other significant findings include the substantial difference in the information dental providers requested vs. the information medical providers shared (Figure 3, Table 3); dental providers having to contact many providers or the same provider multiple times in 45% of consults; and the extended time (longer than 30 days) taken to complete medical consults in 13.8% of the consults (Table 4). The results confirm anecdotal reports regarding inconsistencies in the communications between dental and medical providers; and the additional efforts required by dental providers to obtain information from medical providers. It is also important to note that medical providers did not provide dental providers' requested information in 20% of their responses. These results highlight the critical need to establish appropriate systems and processes to facilitate dental providers' access to their patients' medical information and communicate with medical providers in a timely manner. In the sections below, we discuss our findings and offer recommendations to improve information sharing and care coordination between dental and medical providers.

### Patients' Limited Knowledge of Medication History or Difficulty in Sharing Information Are Significant Barriers

The patient's inability to provide enough medical information to continue with care could have prompted dental providers to seek medical consults. A recent survey of dental providers (21) in our institution reported patients' limited knowledge of their medications, as a significant barrier to obtaining complete medication history. Other studies also suggest that patients, especially older patients with multiple chronic conditions, have difficulty recalling their medical and medication histories (22, 23), thus negatively affecting dental providers' patient care. Medically compromised patients are at higher risk for adverse events due to polypharmacy and multiple co-morbidities that compromise their health. Therefore, it is not always safe to rely on a patient's memory and knowledge to obtain their complete health history. Improving communications and information sharing between dental and medical providers should be a focus of future studies. Health information technology tools such as integrated EDR-EHR and HIE could provide potential solutions to this problem. More detailed discussions are in section 4.5.

### Gaps in Information Dental Providers Requested vs. Information Medical Providers Returned

As shown in Figure 3 and Table 3, several gaps exist between the information dental providers requested and the information medical providers shared. Moreover, medical providers sent complete medical records in a quarter of the consult requests and did not respond to 20% of the consult requests. The results suggest that medical providers either did not understand dental providers' requests or did not have the time to search and share the requested information. However, a survey study conducted by the research team found that dental providers also did not have the time to search through the medical record and locate the relevant information (unpublished data). It is also interesting to note that medical providers shared more information than requested regarding the patients' medication information but did not share information such as medical conditions and specific requests such as ejection fraction information. Future work should investigate the reasons for medical providers not responding to dental providers' requests. Also, future work should develop approaches that allow dental providers to have access to relevant information and utilize medical consults to discuss patients' medical conditions with medical providers. Such approaches will improve care coordination between dental and medical providers, thus enhancing patient care.

### Dental Providers Considered Patients' General Health During Treatment Planning

The percentage of laboratory and diagnostic reports requested by dental providers (Figure 3 and Table 3) and seeking medical providers' recommendations suggest that they considered their patients' general health as an important factor during diagnosis and treatment planning (24). Dental providers' requests for information, such as HbA1C values, blood pressure, CD4 count, and echocardiography reports also demonstrated that they assessed their patient's physical health to undergo dental treatments and to avoid adverse events for the patient or for the dental provider.

### Dental Providers Did Not Always Describe the Patient's Dental Condition/Diagnosis

Although dental providers strived to be specific in their information requests, often, they did not describe their patients' dental condition/diagnosis but described the planned treatment. Even though medical providers are not responsible for dental care, it is important for them to be aware of the patients' dental condition and planned treatment. However, most dental providers currently only use procedure codes during dental care and do not include diagnostic terminologies which are critical for oral disease surveillance, care quality monitoring, and communication with their medical colleagues (25, 26). To encourage dental providers to use standardized diagnostics terminologies, we should include them as part of our dental education curriculum and provide tools to ease the adoption and implementation, such as embedding the terminologies into EDR systems. Moreover, even though not specifically abstracted for this study, it was noted that some abbreviations used were the same for both dental and medical providers, however, several abbreviations have different meanings across the disciplines which risks misinterpretation by one or the other health care provider. Agreement to shared terminologies, such as using the American Society of Anesthesiologists (ASA) physical status classification system (27) to describe patients' status based on their underlining conditions, is urgently needed, especially with the increase in electronic communications and shared databases among all healthcare providers.

### Integrated Electronic Dental Record (EDR)—Electronic Health Record (EHR) Systems and Health Information Exchanges (HIE) as Potential Solutions to Improve Information Sharing and Communication Between Dental and Medical Providers

The inconsistencies, gaps, and delays in sharing information revealed in this study necessitate the need for solutions to improve access to patient medical information for dental providers without overburdening medical providers. Moreover, the mounting evidence pointing to the strong association between oral and overall health has led to increasing calls for integrated EDR-EHR systems to promote information sharing and coordination of care. As a result, large healthcare organizations (HCOs), academic institutions, and federally qualified health centers (FQHCs) have implemented integrated EDR-EHR systems. Dental providers practicing in association with large HCOs have access to patient medical information and have demonstrated improvement in patient care (28, 29). Numerous studies have reported physicians' use of (28, 30–32) integrated EDR-EHR systems to refer patients to dental clinicians and vice versa for preventive and comprehensive care in FQHCs and large HCOs (33, 34). For instance, children enrolled in Medicaid were referred for preventive services such as the fluoride varnish application. Adults diagnosed with diabetes were referred for dental screening and periodontal treatment within the year. Finally, dentists in HealthPartners, an HCO, screened patients for diabetes, blood pressure, oral cancer, and opioid crises and referred them to appropriate medical specialties (35).

However, most dental providers practice in solo and group settings where dental care alone is provided, and these dental practitioners may not have direct access to their patients' medical information. The emergence of community and vendor-supported HIEs have improved medical providers timely access to patient information (36–39). Similarly, for dental providers, access to HIEs might be a solution for improved communications between dental and medical providers and a reduction in the need for dental-medical consults for obtaining patient information. The HIE presents a viable solution to address current information silos and to provide dental providers with timely access to patients' medical information. Enabling dental providers to participate with an HIE will provide real-time access to patient medical history to validate patient-reported information and will reduce the time for dental providers to obtain complete medical information for each patient.

The use of HIEs does not eliminate the medical consult, but rather significantly decreases the need to contact medical providers for information accessible through HIE and improves the quality of the consult questions. These better prepared and more meaningful questions can help to increase the response rate and reduce the response time of the consults. They will also be helpful to get more specific and focused responses from the medical providers.

### Emphasizing the Importance of Interprofessional Collaboration During Dental Care

It is a known fact that the historic divide between dentistry and medicine is a huge barrier to the coordination of care between dental clinicians and medical providers. Chronic conditions such as cardiovascular diseases and obesity are associated with poor oral health and therefore, dental providers must be aware of their patients' underlying conditions. For instance, poorly controlled diabetes is an established risk factor for periodontal disease, and not knowing the patients' status could adversely influence periodontal treatment outcomes as well as healing following tooth extraction, bone grafting, implant placement, and root canal treatments. Besides, people are living longer with multiple chronic conditions that are managed by numerous medications that increase the risk for caries (tooth decay) and other oral diseases. In this study, we found that dental providers asked for recommendations/medical clearance in 39.6% of consults and clarifications regarding medications such as antibiotic prophylaxis and anticoagulant medications in 38.3% of consults (Figure 3, Table 3), even when professional standards and guidelines existed (40, 41). The results denote the importance of interprofessional collaboration and communication to provide optimum dental care and therefore, timely access to information and communication among healthcare professionals can enhance care and reduce costs.

### Strengths and Limitations

This study identified the medical information needs of pre-doctoral dental providers to support continuity of care for their patients. Additionally, despite using a small sample size, an in-depth exploratory analysis was conducted on each medical consult utilizing a minimum of 17 components. Nevertheless, the results regarding the reasons for seeking medical consults are consistent with other studies and related reports (15–17, 21). Like other studies conducted in a single setting, the findings from this study have limited generalizability outside of the IUSD or the academic dental environment. To further explore this study's implications, this study could be repeated and compared among other academic and non-academic institutions.

## Conclusion

The major reasons for dental providers' medical consults are to obtain patient information and seek recommendations/medical clearance for dental care. This study identified that laboratory/diagnostic reports, current medical conditions and medication history, or modification are the major information requests. Also, precautions for dental procedures, antibiotic prophylaxis, and contraindications such as using local anesthesia containing vasoconstrictor are the major advice/recommendations dental providers seek from their medical colleagues. The results highlight the importance of interdisciplinary collaboration between dental and medical providers to provide optimum dental care and the crucial need to have appropriate systems such as integrated EDR-EHR and HIEs to enhance information sharing and communication between dental and medical providers to coordinate patient care.

## Data Availability Statement

The data analyzed in this study is subject to the following licenses/restrictions: The dataset is not publicly available because it contains patient identifiers. Requests to access these datasets should be directed to tpt@iu.edu.

## Author Contributions

JM, KW, and TT contributed to the study design and development of the data abstraction guideline and form. JM, KW, JP, and TG finalized the guideline and reviewed the medical consults. SL, JM, and KW checked the data for accuracy and performed the data analysis. SL, JM, KW, and TT made significant contributions to the drafts of the manuscript. All authors contributed to manuscript revision, read, and approved the submitted version.

## Funding

This study was funded by TT start-up funds through the Indiana University School of Dentistry and in part by the National Library of Medicine (KW) of the National Institutes of Health under Award Number T15NLM012502. The content is solely the responsibility of the authors and does not necessarily represent the official views of the National Institutes of Health or the National Library of Medicine.

## Conflict of Interest

JM who worked in this project as graduate student at the Indiana University (IU) School of Dentistry, is currently employed by ELLKAY LLC. The remaining authors declare that the research was conducted in the absence of any commercial or financial relationships that could be construed as a potential conflict of interest.

## Publisher's Note

All claims expressed in this article are solely those of the authors and do not necessarily represent those of their affiliated organizations, or those of the publisher, the editors and the reviewers. Any product that may be evaluated in this article, or claim that may be made by its manufacturer, is not guaranteed or endorsed by the publisher.
